# Supplementary material for: Structural Basis of Zika Virus Specific Neutralization in Subsequent Flavivirus Infections
Source: Viruses. 2020 Nov 24;12(12):1346. doi: 10.3390/v12121346 (PMC7760643; doi:10.3390/v12121346)
Supplement: Supplementary file 1 [file viruses-12-01346-s001.pdf]

Supplementary information to accompany:

Structural basis of zika virus specific neutralization in subsequent flavivirus infections

Madhumati Sevvana,[1] Thomas F. Rogers,[2] Andrew S. Miller,[1] Feng Long,[1] Thomas Klose,[1] Nathan Beutler,[2] Yen-Chung Lai,[2] Mara Parren,[2] Laura M. Walker,[3] Geeta Buda,[1] Dennis R. Burton,[2,4] Michael G. Rossmann[1] and Richard J. Kuhn,[1,5*] and

Supplementary information consists of:

Figures S1-S7

Tables S1 and S2

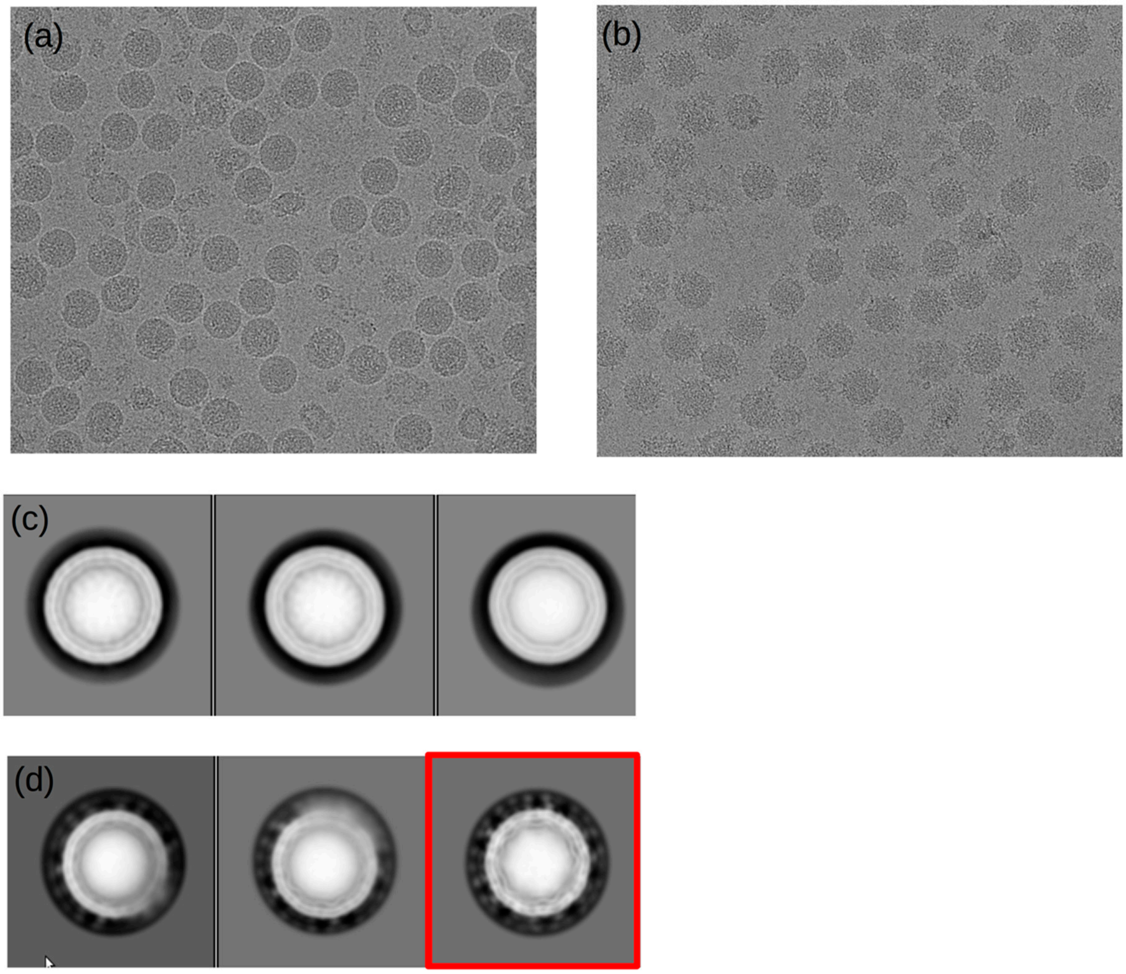

Figure S1. Micrographs and 2D Classifications of Native ZIKV and ZIKV in Complex with ADI-Fab30056

(a) and (c) Native ZIKV micrograph and 2D classification (b) and (d) Micrograph and 2D classification of ZIKV in complex with ADI-Fab30056. The class selected for ZIKV-Fab complex reconstruction is highlighted in red.

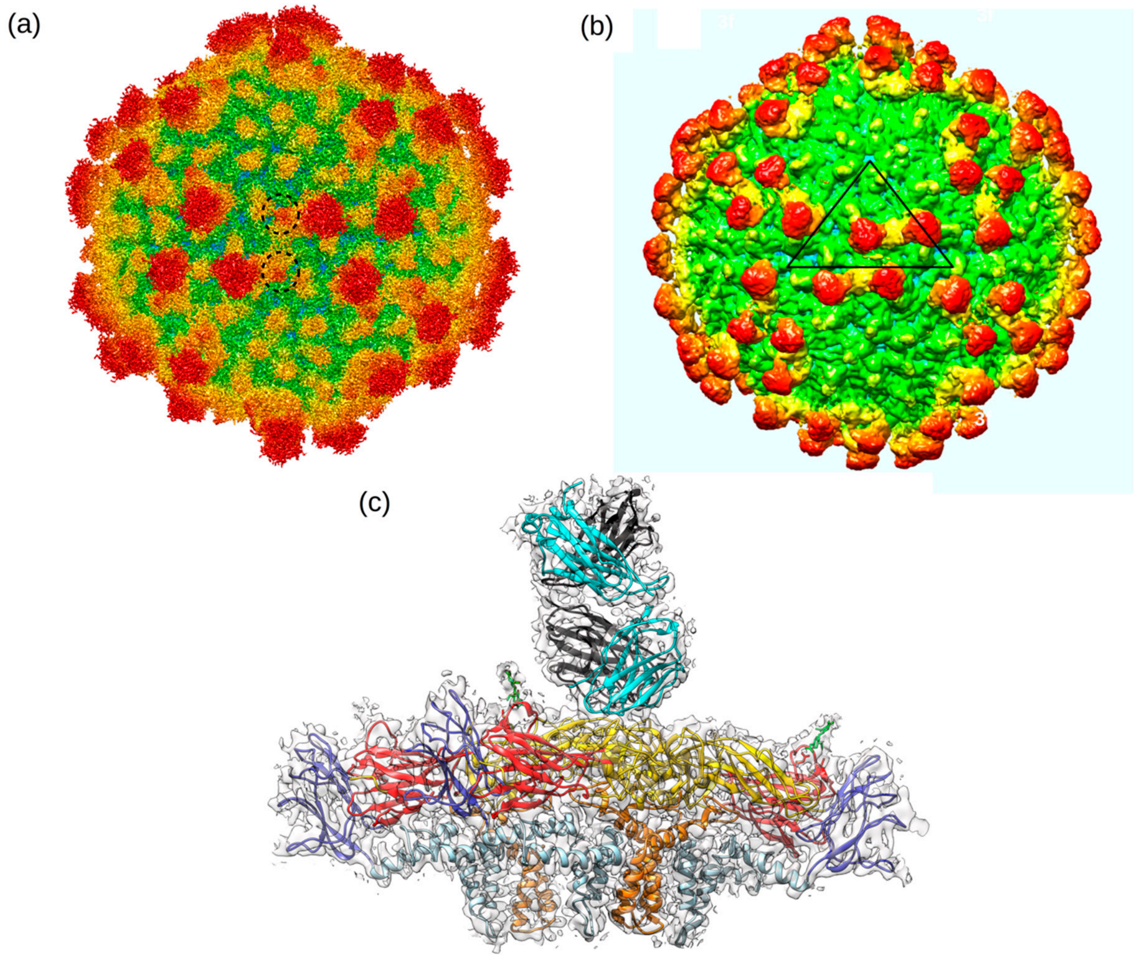

Figure S2. Comparison of Cryo-EM Maps of ZIKV in complex with ADI-30056 and ZIKV-117

(a) ZIKV in complex with ADI-Fab30056 at a resolution of 4.0 Å viewed down an icosahedral 2-fold axis contoured at 1.5 σ, (b) ZIKV in complex with ZIKV117 at a resolution of 6.3 Å, (c) Map contoured at 3 σ around the E proteins and ADI-Fab30056.

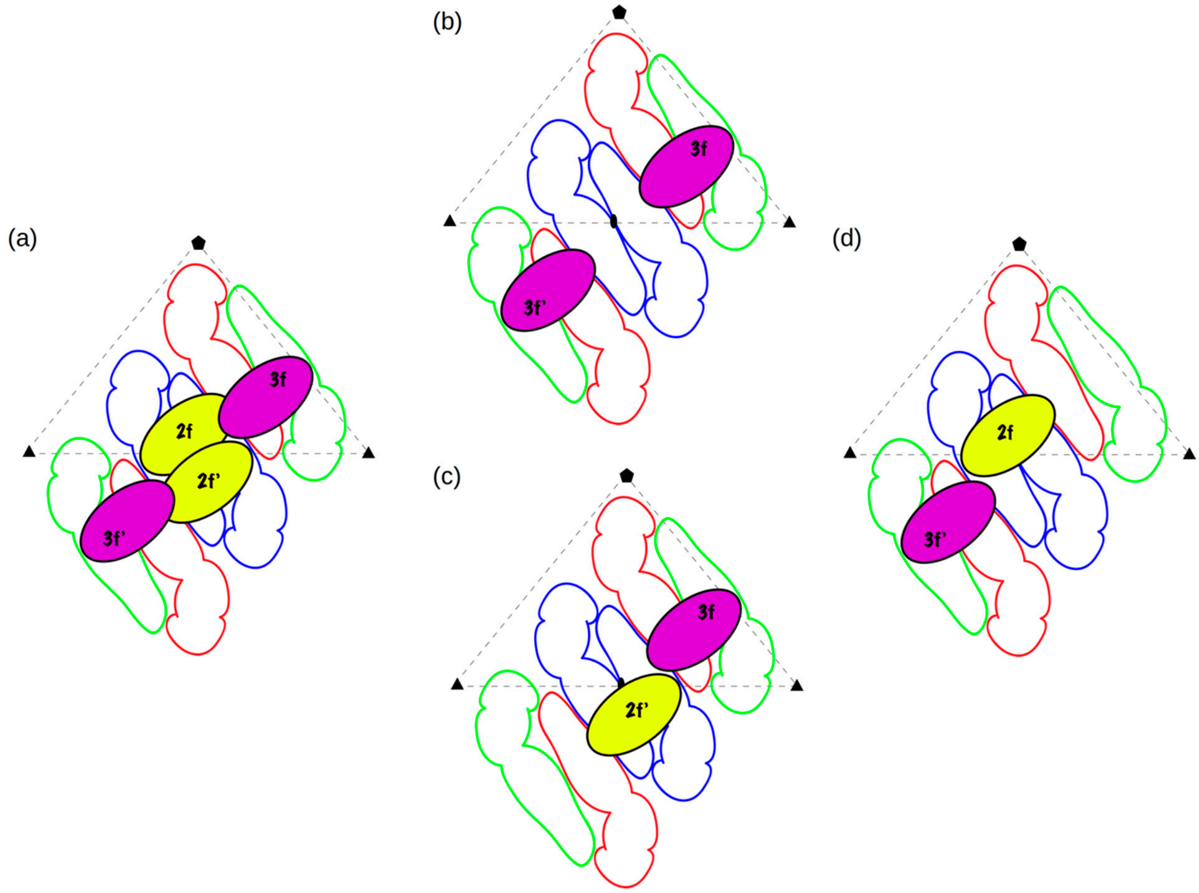

Figure S3. ADI-Fab30056 binding scenarios

(a) Fab binding state on an icosahedrally averaged map of ZIKV in complex with ADI-Fab30056. The icosahedral asymmetric unit in ZIKV consisting of three E monomers, ZE2, ZE5, and ZE3 are colored in blue, red and green, respectively. The Fabs binding near the 2-fold, 3-fold and 5-fold axes are named 2f, 3f and 5f Fabs, respectively. Two adjacent asymmetric units consisting of three E protein dimers form a raft. (b) The 3f-3f' possible Fab binding state. (c) The 3f-2f'possible Fab binding state. (d) The 3f'-2f possible Fab binding state.

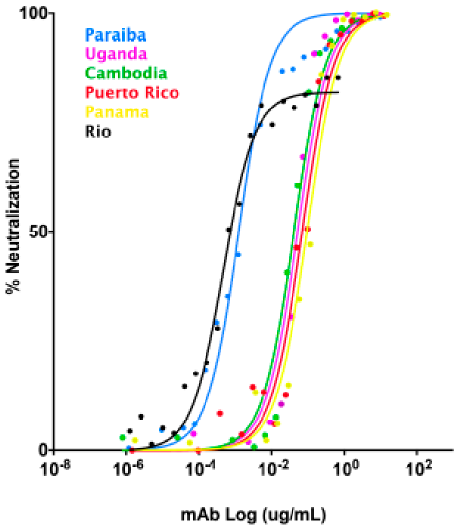

Figure S4. ADI-30056 Binding to ZIKV

Focus reduction neutralization test of different ZIKV strains using ADI-30056 IgG.

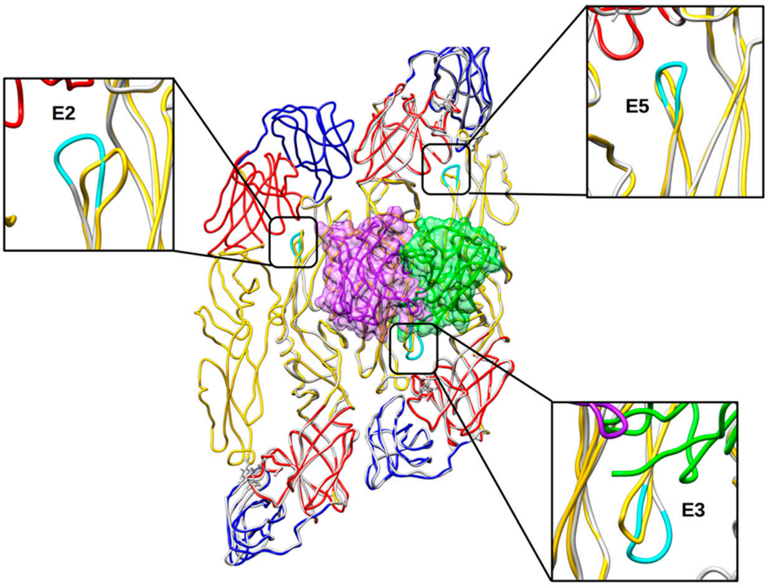

Figure S5. Superposition of ZIKV-ADI-Fab30056 and Native ZIKV Structure

The displaced i-j loop in the uncomplexed ZIKV structure is colored in cyan. The E protein domains I, II and III are colored red, yellow and blue, respectively. The Fab heavy and light chains are colored in magenta and green, respectively.

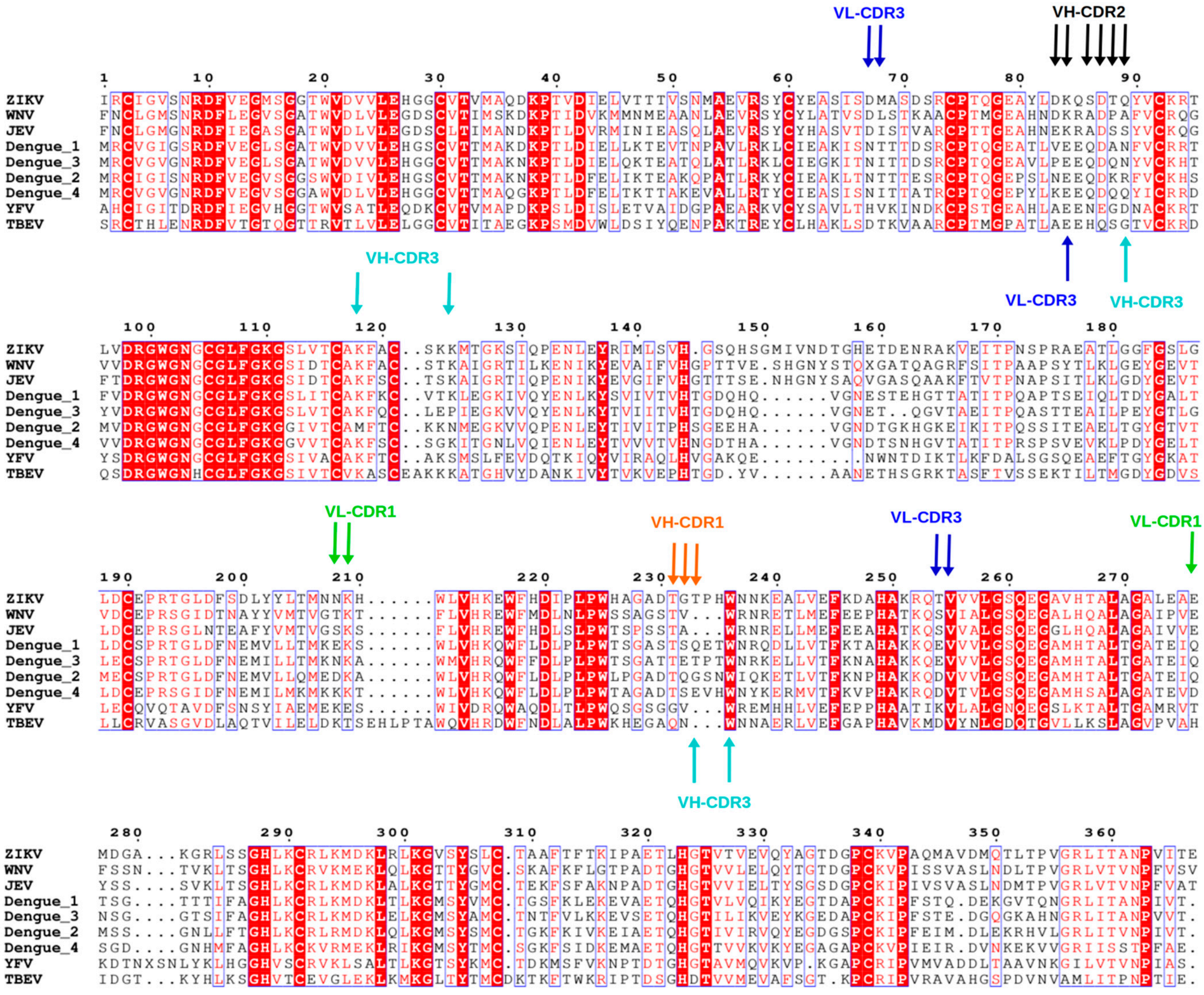

Figure S6. Multiple Sequence Alignment of Different Flavivirus Consensus Sequences

The Fab VH and VL-CDR loop interacting residues are labelled and highlighted using arrows.

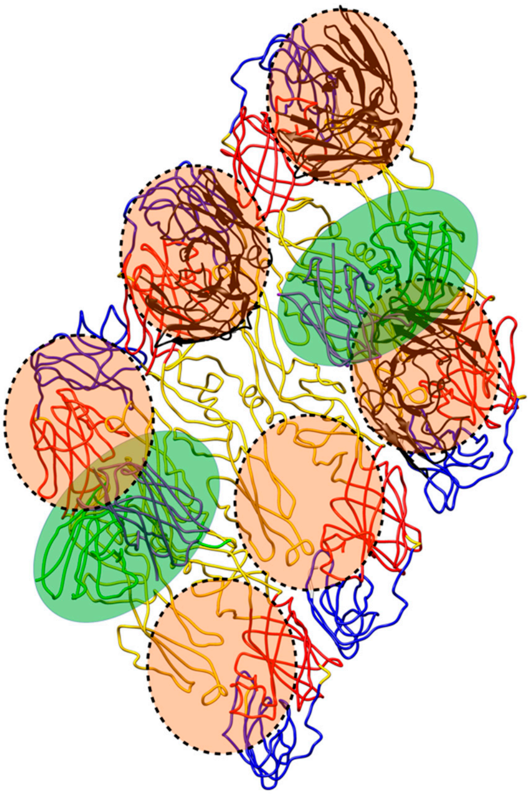

Figure S7. The Accessibility of Ab C10 (orange ovals) upon binding of ADI-Fab30056 at the 3f-3f' position (green ovals) on the raft. Color coding of the E protein is the same as in Figure 1.

Table S1. Sequence variation among flavivirus E proteins at ADI-Fab30056-CDR interacting regions

Fab-V-loops	ZIKV	WNV	JEV	DENV1	DENV2	DENV3	DENV4	YFV
**VH-CDR1**	T231<br>G232<br>T233<br>D83	-<br>-<br>-<br>D	-<br>-<br>-<br>D	T<br>S<br>Q<br>V	T<br>Q<br>G<br>N	T<br>E<br>T<br>P	T<br>S<br>E<br>K	-<br>-<br>-<br>A
**VH-CDR2**	D83<br>K84<br>S86<br>D87<br>T88	D<br>K<br>R<br>D<br>P	E<br>K<br>R<br>D<br>S	V<br>E<br>E<br>D<br>A	N<br>E<br>E<br>D<br>K	P<br>E<br>E<br>D<br>Q	K<br>E<br>E<br>D<br>Q	A<br>E<br>E<br>E<br>G
**VH-CR3**	Q89<br>K118<br>K124<br>T233	A<br>K<br>K<br>-	S<br>K<br>K<br>-	N<br>K<br>K<br>Q	R<br>M<br>N<br>G	N<br>K<br>P<br>T	Q<br>K<br>K<br>E	D<br>K<br>S<br>-
**VL-CDR1**	N208<br>E276	T<br>E	S<br>E	E<br>Q	D<br>Q	N<br>Q	K<br>D	K<br>T
**VL-CDR3**	I65<br>D67<br>M68<br>K84<br>T254<br>V255	V<br>D<br>L<br>K<br>S<br>V	V<br>D<br>I<br>K<br>S<br>V	I<br>N<br>T<br>E<br>E<br>V	L<br>N<br>T<br>E<br>E<br>V	I<br>N<br>T<br>E<br>D<br>V	I<br>N<br>T<br>E<br>D<br>V	L<br>H<br>V<br>E<br>K<br>V

Table S2. CDR loop residues interacting with ZIKV are shown in red font.

**Fab-V-Loops**	**Fab residues**
VH-CDR1	FTFRNFGIH
VH-CDR2	FIRYDGRNKYYADSVKG
VH-CDR3	ARDGEETSPGSFDY
VL-CDR1	RASESVSTVLN
VL-CDR2	AASTLPS
VL-CDR3	QQSYFSPRT
